# The link between long-term nut consumption and health outcomes: A hard nut to crack?

**DOI:** 10.17179/excli2020-2640

**Published:** 2020-08-03

**Authors:** Omid Eslami, Farzad Shidfar

**Affiliations:** 1 Department of Nutrition, School of Public Health, Iran University of Medical Sciences, Tehran, Iran

## ⁯⁯

***Dear Editor,***

In the recent issue of The American Journal of Clinical Nutrition, de Souza and colleagues (2020[7]), in a multinational prospective cohort study, reported an inverse relationship between long-term nut intake and mortality. The results of this study add to the growing body of evidence supporting the favorable effect of nuts on health outcomes. However, it failed to show such a favorable significant association for the risk of major cardiovascular disease (CVD), which was contrary to the previous literature (Becerra-Tomás et al., 2019[1]). The authors attributed this controversy to the variations in the type of nuts consumed as well as the CVD risk markers, and the presence of background CVD risk. 

Although, we believe that another factor that should be taken into the account is the analysis of diet-disease relationship in the context of foods' substitution or addition. The results of two independent prospective analyses from the Nurses' Health Study revealed that substituting one serving of nuts per day instead of an equal amount of red meat was associated with 30 % and 17 % lower risk of coronary heart disease (CHD) and stroke, respectively (Bernstein et al., 2012[2], 2010[3]). Substitutional analysis can provide a better understanding of the diet-disease relationship compared to the additional model, which considers the effect of an independent increase in the dietary exposure of the interest regardless of the other foods in the whole diet (Boeing, 2013[5]). For example, in a prospective cohort study by Daniel et al. (2011[6]), substitution analysis revealed that each 10 g increase in white meat intake instead of the equal amount of red meat was accompanied by a decrease in risk of some gastrointestinal and respiratory cancers; however, in the addition analysis, with the red meat intake was remained stable, the findings were less consistent. Based on this evidence, the authors suggested that the protective effect of white meat on cancer might be mostly due to the substitution with red meat; and simply including white meat in the diet without decreasing red meat might have a less protective effect against the risk of cancer. 

The interpretation of the relationship in the de Souza et al. study is of great importance since it was found that the intake of red and processed meat had a significant increasing trend across the incremental categories of nut intake, and it is likely that addition of nuts into the diet irrespective of reduction in intake of red meat might have mitigated the health benefits of nuts on the risk of CVD and stroke. 

Another interesting finding of this study was a protective effect of nut intake against the risk of mortality in subjects with overweight or obesity (i.e., a body mass index [BMI] ≥ 25 kg/m^2^). While such a significant association was not found for those with a BMI within the normal ranges. This finding has a great implication for public health, since a high BMI, outside the normal ranges, has been directly linked with the risk of total and cardiovascular mortality (Bhaskaran et al., 2018[4]). The relationship between nuts with obesity and related disorders seems to be complex. Incorporating nuts into the diet can substantially increase the dietary energy density (Souza et al., 2015[10]), a factor that has been linked to an increased risk of obesity. Although accumulating evidence from prospective cohort studies and randomized controlled trials has shown an inverse association between nut intake and risk of weight gain, overweight, and obesity (Eslami et al., 2019[8]; Li et al., 2018[9]); this beneficial effect has been attributed to their high nutrient-density, which mainly comprises unsaturated fatty acids, plant protein, fiber, and polyphenols. The finding of this study raises the hypothesis that nuts might exert their protective effect against mortality via a decrease in risk of obesity, as a well-established CVD risk factor. 

In summary, the de Souza et al. study offers valuable clues, which helps us to broaden our perspective regarding the health outcomes of nuts, as a nutrient-rich, energy-dense source.

## Conflict of interest

The authors declare that there are no conflicts of interest.

## Financial support

This research did not receive any specific grant from funding agencies in the public, commercial, or not-for-profit sectors.
